# Effect of Oregano Essential Oil (*Origanum vulgare subsp. hirtum*) on the Storage Stability and Quality Parameters of Ground Chicken Breast Meat

**DOI:** 10.3390/antiox5020018

**Published:** 2016-06-07

**Authors:** Marwan Al-Hijazeen, Eun Joo Lee, Aubrey Mendonca, Dong Uk Ahn

**Affiliations:** 1 Department of Animal Science, Iowa State University, Ames, IA 50011, USA; marwana@mutah.edu.jo (M.A.-H.); duahn@iastate.edu (D.U.A.); 2 Department of Food and Nutrition, University of Wisconsin-Stout, Menomonie, WI 54751, USA; 3 Department of Food Science and Human Nutrition, Iowa State University, Ames, IA 50011, USA; amendon@iastate.edu

**Keywords:** oregano essential oil, cooked chicken breast meat, volatiles, lipid and protein oxidation, color

## Abstract

A study was conducted to investigate the effect of oregano essential oil on the oxidative stability and color of raw and cooked chicken breast meats. Five treatments, including (1) control (none added); (2) 100 ppm oregano essential oil; (3) 300 ppm oregano essential oil; (4) 400 ppm oregano essential oil; and (5) 5 ppm butylated hydroxyanisole (BHA), were prepared with ground boneless, skinless chicken breast meat and used for both raw and cooked meat studies. For raw meat study, samples were individually packaged in oxygen-permeable bags and stored in a cold room (4 °C) for 7 days. For cooked meat study, the raw meat samples were vacuum-packaged in oxygen-impermeable vacuum bags and then cooked in-bag to an internal temperature of 75 °C. After cooling to room temperature, the cooked meats were repackaged in new oxygen-permeable bags and then stored at 4 °C for 7 days. Both raw and cooked meats were analyzed for lipid and protein oxidation, volatiles, and color at 0, 3, and 7 days of storage. Oregano essential oil significantly reduced (*p* < 0.05) lipid and protein oxidation, and improved color stability of raw and cooked meat. However, oregano oil at 400 ppm showed the strongest effect for all these parameters. Hexanal was the major aldehyde, which was decreased significantly (*p* < 0.05) by oregano oil treatment, in cooked meat. Overall, oregano essential oil at 100–400 ppm levels could be a good preservative that can replace the synthetic antioxidant in chicken meat.

## 1. Introduction

Poultry meat is among the most popular meats in the world because of its low price, short production time, and ease of preparation [1]. Chicken meat contains high levels of polyunsaturated fatty acids, which make it susceptible to oxidative deterioration during storage [2]. Both raw and cooked poultry meats can be oxidized, but cooked meat is more susceptible to oxidative changes than raw meat [3,4]. Lipid oxidation produces many volatile compounds responsible for off-flavor and rancidity in meat [5]. Other quality characteristics such as color and texture of meat can also be influenced by the oxidation of meat [6,7]. Several synthetic antioxidants such as butylated hydroxyanisole (BHA), butylated hydroxytoluene (BHT), and propyl gallate (PG) have been used widely in the food industry to prevent the deteriorative changes. However, the use of synthetic antioxidants is discouraged due to consumer concerns about their negative effects on human health. Therefore, the food industry is interested in finding new natural antioxidant sources to replace the synthetic ones [8].

Herbs, spices and many plant extracts have been widely used in foods to improve their flavor and quality, and to extend their shelf-life [9]. Oregano essential oil is one of the many plant extracts that have strong antioxidant effects when added to meat [10,11,12]. The antioxidant effect of oregano essential oil is due to its high polyphenol contents; Carvacrol, thymol, *p*-cymene and γ-terpenene are the major components responsible for the antioxidant activity of oregano essential oil [13,14,15].

Previous research works of oregano essential oil were focused on adding the oil directly to the diets of poultry [16,17], lamb [18], and swine [19] to stabilize the meats from those animals. However, few works have been done to determine the antioxidant effects of oregano essential oil directly added to meat [1,10,12]. Many previous works showed that adding oregano essential oil directly to the meat products had positive effects on meat quality. Some research investigated the combination effects of carvacrol and thymol on the poultry meat patties and showed positive effects on their color and reduced off-odor formation [20]. Sullivan *et al.* [21] studied the effect of natural antioxidants such as rosemary, sage and tea catechins on the chicken nugget and found that the addition of these antioxidants decreased lipid oxidation, and improved color and storage stability of the product. Keokamnerd *et al.* [22] studied the effects of adding commercial rosemary oleoresin on ground chicken meats, which were packaged in a high-oxygen atmosphere, and found positive effects on raw meat appearance and cooked meat flavor during storage. Oxidation process was slower, and the color was more stable in ground meat when rosemary oleoresin was added. Oregano essential oil, which contains high levels of carvacrol as the major antioxidant, showed higher antioxidant activity than other herb extracts [10,23]. Even though oregano essential oil has a great potential as a natural antioxidant, little is known about the optimal level to prevent oxidative changes of meat products while maintaining other quality parameters such as flavor.

The objective of this study was to investigate the effect of different levels of oregano essential oil on major meat quality parameters, including oxidative stability and color of raw and cooked ground chicken breast meat.

## 2. Materials and Methods

**Sample preparation:** One hundred and twenty, 6-week-old broilers raised on a corn-soybean meal diet were slaughtered using the USDA guidelines [24]. The chicken carcasses were chilled in ice water for 2 h, drained in a cold room, and the breast muscles were separated from the carcasses 24 h after slaughter. The breast muscles were ground twice through a 10-mm and a 3-mm plates (Kitchen Aid, Inc., St. Joseph, MI, USA) after removing skins before use. Five different treatments, including (1) control (none added); (2) 100 ppm oregano essential oil; (3) 300 ppm oregano essential oil; (4) 400 ppm oregano essential oil; and (5) 5 ppm butylated hydroxyanisole (BHA), were prepared. The oregano essential oil was produced from a certified company in Turkey (Healthy-Health, Staten Island, NY, USA), which contains 80.12% carvacrol. BHA powder (0.1 g) and oregano essential oil (1.25 g) were dissolved in 10 mL of 100% ethanol, and then mixed with 50 mL mineral oil to make their stock solutions. The ethanol added was removed using a rotary evaporator (BUCH Rotavapor, Model R-200, BUCHI Co., New Castle, DE, USA) at 70 °C, 175 mbar vacuum pressure before adding the stock solution to meat samples. Each treatment was added to the ground breast meat and then mixed for 2 min (speed set at 2) in a bowl mixer (Model KSM 90; Kitchen Aid Inc., St. Joseph, MI, USA). The control treatment was added with the same amounts of mineral oil to provide the same conditions.

For raw-meat study, the prepared meat samples (approximately 100 g each) were individually packaged in oxygen-permeable bags (polyethylene, 4 × 6.2 mil, Associated Bag Co., Milwaukee, WI, USA), stored in a cold room at 4 °C for 7 days, and analyzed for lipid and protein oxidation, and color at 0, 3, and 7 days of storage.

For cooked-meat study, the raw meat samples (approximately 100 g each) were vacuum-packaged in oxygen-impermeable vacuum bags (O_2_ permeability, 9.3 mL O_2_/m^2^/24 h at 0 °C, Koch, Kansas City, MO, USA) first, and then the meats were cooked in-bag in a 90 °C water bath (Isotemp^®^, Fisher Scientific Inc., Pittsburgh, PA, USA) until the internal temperature of the meat reached to 75 °C. After cooling to room temperature, the cooked meats were repackaged individually in new oxygen-permeable bags (polyethylene, 4 × 6.2 mil, Associated Bag Co., Milwaukee, WI, USA), stored in a cold room at 4 °C for 7 days, and analyzed for lipid and protein oxidation, color, and volatiles at 0, 3, and 7 days of storage as same as in the raw-meat study. 

**2-Thiobarbituric acid-reactive substances (TBARS) measurement:** Lipid oxidation was determined using a TBARS method [25]. Ground chicken meat (5g), 15 mL of deionized distilled water (DDW), and 50 μL BHT (7.2%) were added to a 50-mL test tube, and homogenized using a Polytron (Type PT 10/35, Brinkman Instruments Inc., Westbury, NY, USA) for 15 s at high speed. The meat homogenate (1 mL) and thiobarbituric acid/trichloroacetic acid solution (15 mM TBA/15% TCA, 2 mL) were vortex-mixed and incubated in a boiling water bath for 15 min to develop color. After cooling for 10 min in ice-water, the samples were mixed again, and then centrifuged for 15 min at 2500× *g* at 4 °C. The supernatant was collected and the absorbance read at 532 nm against a blank containing 1 mL of DDW and 2 mL of TBA/TCA solution. The TBARS values were expressed as mg of malondialdehyde (MDA) per kg of meat.

**Color measurement:** The color of meat was measured on the surface of meat samples using a Konica Minolta Color Meter (CR-410, Konioka Minolta, Osaka, Japan). The color meter was calibrated using an illuminant source C (Average daylight) on a standard white ceramic tile covered with the same packaging film as the ones used for meat samples to negate the color and light reflectance properties of the packaging material. The color was expressed as CIE L*-(lightness), a*-(redness), and b*-(yellowness) values [26]. The areas selected for color measurement were free from obvious defects that may affect the uniform color readings. An average of two random readings on the top of sample surface was used for statistical analysis.

**Volatile analysis:** Volatiles of samples were analyzed using a Solatek-72 Multimatrix-Vial Auto-sampler/Sample Concentrator 3100 (Tekmar-Dohrmann, Cincinnati, OH, USA) connected to a GC/MS (Model 6890/5973; Hewlett-Packard Co., Wilmington, DE, USA) according to the method of Ahn *et al.* [27]. Sample (3 g for raw meat and 2 g for cooked meat) was placed in a 40-mL sample vial, flushed with helium gas (40 psi) for 3 s, and then capped airtight with a Teflon-fluorocarbon resin/silicone septum (I-Chem Co., New Castle, DE, USA). Samples from different treatment were randomly organized on the refrigerated (4 °C) holding try to minimize the variation of the oxidative changes among treatments during analysis. The meat sample was purged with helium (40 mL/min) for 14 min at 20 °C. Volatiles were trapped using a Tenax/charcoal/silica column (Tekmar-Dohrmann) and desorbed for 2 min at 225 °C, focused in a cryofocusing module (−70 °C), and then thermally desorbed into a capillary column for 2 min at 225 °C. An HP-624 column (7.5 m, 0.25 mm i.d., 1.4 μm nominal), an HP-1 column (52.5 m, 0.25 mm i.d., 0.25 μm nominal), and an HP-Wax column (7.5 m, 0.250 mm i.d., 0.25 μm nominal) were connected using zero dead-volume column connectors (J & W Scientific, Folsom, CA, USA). Ramped oven temperature was used to improve volatile separation. The initial oven temperature of 25 °C was held for 5 min. After that, the oven temperature was increased to 85 °C at 40 °C per min, increased to 165 °C at 20 °C per min, and then increased to 230 °C at 5 °C per min and held for 2.5 min at the temperature. Constant column pressure at 22.5 psi was maintained. The ionization potential of MS was 70 eV, and the scan range was 20.1 to 350 m/z. The identification of volatiles was achieved by the Wiley Library (Hewlett-Packard Co., Wilmington, DE, USA). The area of each peak was integrated using ChemStation™ software (Hewlett-Packard Co., Wilmington, DE, USA), and the total peak area (total ion counts × 10^4^) was reported as an indicator of volatiles generated from the samples.

**Protein oxidation (total carbonyl):** Protein oxidation was determined by the method of Lund *et al.* [28] with minor modifications. One gram of meat sample was added with 10 mL of pyrophosphate buffer (2.0 mM Na_4_P_2_O_7_, 10 mM Trizma-maleate), 100 mM KCl, 2.0 mM MgCl_2_, and 2.0 mM ethylene glycol tetraacetic acid, pH 7.4) and homogenized using a Brinkman Polytron (Type PT 10/35). Two equal amounts of meat homogenate (2 mL) were taken from a sample, precipitated with 2 mL of 20% trichloroacetic acid, and centrifuged at 12,000× *g* for 5 min at room temperature. After centrifugation, one of the pellet was dissolved with 2 mL of 10 mM 2,4-dinitrophenylhydrazine in 2 M HCl and the other one was dissolved with 2 M HCl (blank), and were incubated for 30 min in the dark. During the incubation, the samples were vortex-mixed for 10 s every 3 min. The protein was further precipitated with 2 mL of 20% trichloroacetic acid and centrifuged at 12,000× *g* for 5 min. The 2,4-dinitrophenylhydrazine was removed by washing the pellets 3 times with 4 mL of 10 mM HCl in 1:1 (vol/vol) ethanol:ethyl acetate, followed by centrifuging at 12,000× *g* for 5 min. The pellets were finally solubilized in 2 mL of 6.0 mM guanidine hydrochloride dissolved in 20 mM potassium dihydrogen phosphate (pH = 2.3). The samples were kept at 5 °C overnight and centrifuged to remove insoluble materials. The absorbance of supernatants was read at 370 nm and the value of blank sample was subtracted from their corresponding sample value. The protein concentration of meat samples was measured using Protein Assay Kit (Bio-Rad Laboratories, Hercules, CA, USA) following Microplate Assay protocol at 280 nm (BioTek-Gen5 Microplate data collection and analysis software/BioTek Instruments, Inc., Model S4MLFPTA, Winooski, VT, USA). The carbonyl content was calculated as nmol/mg protein using absorption coefficient of 22,000/M/cm as described by Levine *et al.* [29].

**Statistical analysis**: Data were analyzed using the procedures of the generalized linear model (Proc. GLM, SAS program, Version 9.3, 2012, SAS Institute Inc., Cary, NC, USA) [30]. Mean values and standard error of the means (SEM) were reported. The significance was defined at *p* < 0.05 and Tukey test or Tukey’s Multiple Range test were used to determine whether there are significant differences between the mean values.

## 3. Results and Discussion

**Lipid Oxidation:** Lipid oxidation is among the most critical quality parameters because it not only influences protein oxidation [31] that causes meat discoloration [32], but also is primarily responsible for producing many off-odor and rancid flavor in the meat during storage [33,34]. The antioxidant activity of oregano essential oil indicated that there were no significant differences (*p* > 0.05) among treatments at day 0 in raw meat. TBARS values increased more in the cooked meat compared to the raw meat during storage. In general, adding oregano essential oil to both raw and cooked ground chicken meats reduced the TBARS values (Table 1). This result agreed with other studies that added oregano oil directly to the meat [1,10] or indirectly in the animal diets [16,35]. Among the treatments, oregano essential oil at 400 ppm showed the highest antioxidant effect. The use of higher levels of oregano oil than 400 ppm can further improve its antioxidant effect, but may negatively affect the sensory characteristics of meat [36]. Cooked meat was more sensitive to oxidative changes than the raw meat because the antioxidant enzymes in meat were denatured during cooking, iron ions released from the intracellular to extracellular compartment, and membrane bi-layers became damaged, and phospholipids were open to catalysts and oxygen during cooking and storage [3]. However, the TBA values for the ground raw chicken meat showed little changes during storage regardless of the oregano essential oil treatments. The raw meat values are in agreement with Chouliara *et al.* [1] who reported TBARS values of 0.1–0.28 in fresh breast chicken meat when 0.1% oregano oil was added and stored for 7 days. Kim *et al.* [37] reported TBARS values of 0.13–0.68 for raw turkey meat after 7 days of storage. All the oregano essential oil levels (100, 300, and 400 ppm) showed significantly lower TBARS values compared with the control for both raw and cooked meat after 7 days of storage. The oregano essential oil at 400 ppm, however, showed stronger effect than BHA (5 ppm), especially on the cooked meat at Day 7 (Table 1). The oregano essential oil at 100 ppm and 200 ppm were the minimum levels that could be used to slow down the oxidative changes in ground chicken meat.

**Protein oxidation:** The functional properties of proteins such as protein solubility, gelation, and emulsification capacity in the foods are dependent on their amino acid composition and structure. The relationship between protein oxidation and overall food quality is also well documented [38,39]. Ground meat is more susceptible to oxidation than the whole meat cuts due to their size and surface area that contact with oxygen. Several other factors that may increase protein carbonyl formation in the meat system include metal catalysts (iron, copper, hem and non-heme iron, and myoglobin), pH, temperature, and presence of other inhibitors (antioxidant phenolic compounds) [39,40,41,42]. In this study, oregano essential oil significantly (*p* < 0.05) reduced the total carbonyl formation (nmol/mg of protein), especially in the cooked meat (Table 2). There was no significant difference (*p* > 0.05) in the total carbonyl content between the treatments in raw meat during the first 3 days of storage. The changes of total carbonyl content in the raw meat compared to the cooked meat during storage were very low because the oxidative processes in the cooked meat were faster than in the raw meat. In addition, the total antioxidant capacity of the fresh chicken meat is usually higher than that of the cooked meat. The reduction of antioxidant capacity in cooked meat is mainly due to the denaturation of antioxidant enzymes and the loss of endogenous antioxidants by heat [10,43]. Generally, the formation of total carbonyl agrees with the TBARS values in raw meat during storage. Oregano essential oil at 400 ppm showed the strongest effect in reducing the total carbonyl values (Table 2). The total estimated carbonyl contents fall in the range of 1–3 nmol/mg protein for raw meat and up to 5 nmol/mg protein for cooked meat products [41,44,45,46]. However, the result from the DNPH method from several studies varied widely (0.5–4.8 nmol/mg protein) depending on the meat type, animal species, experimental conditions, and analysis methods, which make it difficult to give a general conclusion. The total carbonyl values in this study agreed well with the TBARS results, and meats with the highest level of oregano oil produced the lowest total carbonyls (Table 2). The result of this study agreed with Fasseas *et al.* [10] who used oregano essential oil (3% w/w) in ground porcine and bovine raw and cooked meats. They found that the oregano essential oil showed higher antioxidant activity than the sage and control treatments during the storage at 4 °C. There were no significant differences between oregano oil treatments at 100 and 300 ppm, and BHA for both raw and cooked meat at Day 7 (Table 2). The antioxidant effect of oregano essential oil was more prominent in the cooked meat than in the raw meat.

**Color values:** Color is one of the most important quality parameters to determine the consumer’s decision for purchasing meat [47,48,49]. The lightness (L*-values) of ground chicken meat significantly decreased during storage. Oregano essential oil at level 400 ppm showed the highest stabilizing effects for L*- and a*-values (Table 3). However, no significant difference (*p* > 0.05) among the treatments on the redness (a*-values) during storage was detected. All oregano oil treatments showed significant positive effects (*p* < 0.05) on L*-values compared to the control. These results are similar to those of Chouliara *et al.* [1] and Mastromatteo *et al.* [20] who reported that oregano oil or their components had no significant effect on a*-value but significantly preserved the L*-value at the end of storage. In general, b*-values decreased during storage time, but oregano oil maintained the b*-values of the meat after 7 days of storage, which agreed with the results of Choulira *et al.* [1]. There were no significant differences on a*-values between the treatments at day 7 of storage due to the low myoglobin content in the chicken breast meat (0.05 mg/g). Ahn and Lee [50] also reported that a*-values did not change much during 15 days of storage in both aerobic- and vacuum-packaged turkey breast meat. 

**Volatiles production:** Adding oregano essential oil reduced the amount of off-odor volatiles in cooked chicken meat (Table 4, Table 5 and Table 6). Little works were done to determine the effect of oregano oil on the volatiles (ketone, aldehydes, hydrocarbon and sulfur compounds) formation in chicken meat. The volatiles produced by the raw meat were very low and no sulfur or aldehyde compounds detected during storage time. Heptanal, dimethyl disulfide, and 1-butanol were found only in the control at day 0 of storage, indicating the development of lipid oxidation in the raw meat before cooking in the control was minor. In the cooked meat, oregano essential oil at 100 ppm and 300 ppm significantly (*p* < 0.05) reduced the amounts of lipid oxidation-dependent volatiles, such as aldehydes (e.g., propanal, hexanal, pentanal and heptanal) and hydrocarbons (cyclohexane, hexane, heptane and octane), compared to the control. Oregano oil at 400 ppm showed the strongest effect in suppressing the aldehydes formation. Hexanal and pentanal were reported as the major indicators of lipid oxidation [25,51]. In addition, positive relationships between aldehydes and TBARS values were reported [52]. Hexanal formation in the cooked meat increased rapidly during storage (Table 4, Table 5 and Table 6), which reflected the relationship between total aldehydes and lipid oxidation status of cooked chicken meat. The volatile content of aerobically-packaged meat increased more rapidly than the vacuum-packaged ones during storage, indicating the role of oxygen in the formation of volatiles [7].

Sulfur volatiles decreased after 3 days of storage in the control. This result agreed with several studies done using aerobically-stored meats, from which most of the sulfur volatile compounds escaped or disappeared after 5 days of storage [4] due to their high volatility [53,54]. Oregano essential oil at 300 and 400 ppm reduced the amount of dimethyl disulfide at Days 0 and 3 of cooked control meat (Table 4 and Table 5). Minor compounds such as carbon disulfide, dimethyl sulfide, 2-butanone, ethyl acetate, and ethyl propionate, which were not detected consistently, were not shown in the tables. Samples from oregano essential oil treatments showed some terpenoids such as sabinene, *p*-cymene, limonene, camphene, γ-terpenene, β-myrcene, and α-terpenene in varying amounts. These compounds have a significant impact to the odor and flavor of meat products. No carvacrol and thymol were detected from oregano treatment due to their low volatilities.

## 4. Conclusions

Oregano essential oil, at 300 and 400 ppm levels, showed the highest antioxidant effect to preserve the ground chicken meat. Adding >100 ppm of oregano essential oil improved the color stability of raw meat and decreased off-odor volatiles in cooked meat. However, the effects of oregano essential on the lipid and protein oxidation, and the volatiles of cooked meat were more significant than in raw meat. Overall, oregano essential oil can be used in place of synthetic antioxidant (e.g., BHA) to prevent quality deterioration in raw and cooked meat during storage.

## Figures and Tables

**Table 1:** 2-Thiobarbituric acid-reactive substances (TBARS) values of raw and cooked chicken breast meat with different levels of oregano oil during storage.

Time	Control (None)	100 ppm Oregano	300 ppm Oregano	400 ppm Oregano	5 ppm BHA	SEM
*Raw Meat*	TBARS (mg MDA/kg meat)					
Day 0	0.13 ^a,z^	0.12 ^a,y^	0.11 ^a,y^	0.11 ^a,x^	0.13 ^a,x^	0.01
Day 3	0.16 ^a,y^	0.14 ^b,y^	0.12 ^c,xy^	0.12 ^c,x^	0.13 ^bc,x^	0.01
Day 7	0.23 ^a,x^	0.15 ^b,x^	0.13 ^b,x^	0.12 ^b,x^	0.14 ^b,x^	0.01
SEM	0.01	0.01	0.01	0.01	0.01	
*Cooked Meat*	TBARS (mg MDA/kg meat)					
Day 0	0.26 ^a,y^	0.14 ^b,y^	0.09 ^b,y^	0.08 ^b,z^	0.11 ^b,z^	0.01
Day 3	2.44 ^a,x^	2.35 ^a,x^	0.72 ^c,x^	0.27 ^d,y^	1.87 ^b,y^	0.06
Day 7	2.55 ^a,x^	2.43 ^b,x^	0.74 ^d,x^	0.35 ^e,x^	2.02 ^c,x^	0.03
SEM	0.07	0.04	0.02	0.01	0.03	

^a–e^ Values with different letters within a row are significantly different (*p* < 0.05). *n* = 4; ^x–z^ Values with different letters within a column are significantly different (*p* < 0.05). Abbreviation: SEM, standard error of the mean; BHA, butylated hydroxyanisole; TBARS, 2-thiobabituric acid reactive substances; MDA, malondialdehyde.

**Table 2:** Protein oxidation of raw and cooked chicken breast meat with different levels of oregano oil during storage.

Time	Control (None)	100 ppm Oregano	300 ppm Oregano	400 ppm Oregano	5 ppm BHA	SEM
*Raw Meat*	Carbonyl content (nmole/mg protein)					
Day 0	0.70 ^a,y^	0.69 ^a,x^	0.69 ^a,x^	0.65 ^a,x^	0.69 ^a,x^	0.04
Day 3	0.77 ^a,xy^	0.75 ^a,x^	0.74 ^a,x^	0.65 ^a,x^	0.75 ^a,x^	0.05
Day 7	0.94 ^a,x^	0.79 ^ab,x^	0.77 ^b,x^	0.69 ^b,x^	0.80 ^ab,x^	0.03
SEM	0.05	0.04	0.04	0.04	0.06	
*Cooked Meat*	Carbonyl content (nmole/mg protein)					
Day 0	0.69 ^a,y^	0.44 ^b,y^	0.43 ^b,x^	0.40 ^b,x^	0.47 ^b,y^	0.04
Day 3	1.25 ^a,y^	0.50 ^b,y^	0.45 ^b,x^	0.42 ^b,x^	0.48 ^b,y^	0.04
Day 7	1.90 ^a,x^	0.95 ^bc,x^	0.56 ^bc,x^	0.44 ^c,x^	1.00 ^b,x^	0.12
SEM	0.15	0.07	0.06	0.05	0.03	

^a–c^ Values with different letters within a row are significantly different (*p* < 0.05). *n* = 4; ^x,y^ Values with different letters within a column are significantly different (*p* < 0.05). Abbreviation: SEM, standard error of the mean; BHA, butylated hydroxyanisole.

**Table 3:** CIE Color values of raw chicken breast meat with different levels of oregano oil during storage at 4 °C.

Time	Control (None)	100 ppm Oregano	300 ppm Oregano	400 ppm Oregano	5 ppm BHA	SEM
L*-value						
Day 0	64.47 ^ab,x^	64.40 ^ab,x^	64.35 ^b,x^	64.82 ^ab,x^	64.91 ^a,x^	0.12
Day 3	63.49 ^a,y^	63.74 ^a,xy^	63.84 ^a,y^	64.04 ^a,x^	63.89 ^a,y^	0.15
Day 7	62.71 ^b,z^	63.64 ^ay^	63.74 ^a,y^	63.95 ^a,x^	63.67 ^a,y^	0.18
SEM	0.14	0.14	0.09	0.23	0.13	
a*-value						
Day 0	8.65 ^a,x^	8.62 ^a,x^	8.55 ^a,x^	8.62 ^a,x^	8.59 ^a,x^	0.10
Day 3	6.36 ^a,y^	6.35 ^a,y^	6.39 ^a,y^	6.50 ^a,y^	6.38 ^a,y^	0.09
Day 7	6.16 ^a,y^	6.26 ^a,y^	6.39 ^a,y^	6.48 ^a,y^	6.37 ^a,y^	0.11
SEM	0.07	0.16	0.10	0.08	0.06	
b*-value						
Day 0	20.87 ^c,x^	21.07 ^cb,x^	21.27 ^b,x^	21.85 ^a,x^	21.70 ^a,x^	0.09
Day 3	18.90 ^bc,y^	19.01 ^c,y^	19.75 ^bc,y^	21.41 ^a,y^	20.80 ^ab,y^	0.28
Day 7	18.62 ^b,y^	18.58 ^b,y^	18.68 ^a,y^	19.29 ^a,z^	18.97 ^ab,z^	0.10
SEM	0.15	0.14	0.28	0.08	0.16	

^a–c^ Values with different letters within a row are significantly different (*p* < 0.05). *n* = 4; ^x–z^ Values with different letters within a column are significantly different (*p* < 0.05). Abbreviation: SEM, standard error of the mean; BHA, butylated hydroxyanisole.

**Table 4:** Profile of volatiles from cooked chicken breast meat with different levels of oregano oil at day 0.

Compounds	Control (None)	100 ppm Oregano	300 ppm Oregano	400 ppm Oregano	5 ppm BHA	SEM
	Total ion counts × 10^4^					
2-Propanone	2758 ^b^	2552 ^b^	2466 ^b^	2733 ^b^	6001 ^a^	517
1-Butanol	548 ^a^	0 ^b^	18 ^b^	0 ^b^	0 ^b^	36
2-Propanol	381 ^bc^	319 ^c^	1371 ^a^	959 ^ab^	738 ^bc^	133
Hexane	1775 ^a^	336 ^b^	244 ^b^	243 ^b^	169 ^b^	203
Heptane	102 ^a^	59 ^ab^	12 ^b^	49 ^ab^	0 ^b^	19
1-Propanol	2832 ^a^	2622 ^a^	1542 ^b^	1027 ^b^	1720 ^b^	174
Pentanal	215 ^a^	270 ^a^	125 ^a^	292 ^a^	110 ^a^	82
Heptanal	35 ^a^	0 ^b^	0 ^b^	0 ^b^	0 ^b^	2
Octane	86 ^a^	235 ^a^	406 ^a^	319 ^a^	165 ^a^	90
Cyclohexane	107 ^a^	63 ^abc^	86 ^ab^	30 ^bc^	0 ^c^	17
Dimethy disulfide	507 ^a^	0 ^b^	0 ^b^	0 ^b^	0 ^b^	82
Hexanal	467 ^a^	167 ^b^	93 ^b^	91 ^b^	551 ^a^	66
α-Pinene	0 ^b^	265 ^a^	226 ^a^	362 ^a^	0 ^b^	36
Camphene	0 ^b^	256 ^ab^	457 ^ab^	715 ^a^	0 ^b^	142
Limonene	0 ^b^	72 ^ab^	173 ^a^	165 ^a^	0 ^b^	25
β-Myrcene	0 ^c^	162 ^bc^	1056 ^ab^	1891 ^a^	0 ^c^	226
γ-Terpenene	0 ^b^	285 ^b^	2159 ^ab^	4637 ^a^	0 ^b^	682
Sabinene	0 ^c^	0 ^c^	149 ^b^	354 ^a^	0 ^c^	20

^a–c^ Different letters within a row are significantly different (*p* < 0.05). *n* = 4. Abbreviation: SEM, standard error of the mean; BHA, butylated hydroxyanisole.

**Table 5:** Profile of volatiles from cooked chicken breast meat with different levels of oregano oil at day 3.

Compounds	Control (None)	100 ppm Oregano	300 ppm Oregano	400 ppm Oregano	5 ppm BHA	SEM
	Total ion counts × 10^4^					
2-Propanone	4106 ^a^	4854 ^a^	4530 ^a^	4517 ^a^	3458 ^a^	797
Pentane	580 ^a^	0 ^b^	0 ^b^	0 ^b^	0 ^b^	61
2-Propanol	620 ^b^	3324 ^ab^	3643 ^a^	1337 ^ab^	905 ^ab^	668
Hexane	216 ^a^	20 ^b^	0 ^b^	0 ^b^	0 ^b^	15
Heptane	86 ^a^	0 ^b^	0 ^b^	0 ^b^	0 ^b^	7
1-Propanol	2279 ^a^	1299 ^b^	2136 ^a^	1956 ^a^	1703 ^ab^	134
Propanal	58 ^b^	15 ^b^	0 ^b^	0 ^b^	144 ^a^	14
Pentanal	1065 ^a^	174 ^bc^	126 ^bc^	0 ^c^	466 ^b^	89
Heptanal	0	0	0	0	0	0
Octane	147 ^a^	204 ^a^	160 ^a^	175 ^a^	179 ^a^	43
Cyclohexane	31 ^a^	0 ^b^	0 ^b^	0 ^b^	0 ^b^	5
Dimethy disulfide	23 ^a^	0 ^b^	0 ^b^	0 ^b^	0 ^b^	1
Hexanal	11371 ^a^	3194 ^b^	1341 ^b^	1448 ^b^	4167 ^b^	752
α-Pinene	0 ^b^	0 ^b^	475 ^a^	444 ^a^	0 ^b^	51
Camphene	0 ^b^	0 ^b^	299 ^a^	328 ^a^	0 ^b^	55
Limonene	0 ^b^	0 ^b^	381 ^a^	430 ^a^	0 ^b^	92
β-Myrcene	0 ^b^	0 ^b^	577 ^ab^	740 ^a^	0 ^b^	163
γ-Terpenene	0 ^b^	0 ^b^	1609 ^ab^	1984 ^a^	0 ^b^	396
Sabinene	0 ^b^	0 ^b^	180 ^a^	315 ^a^	0 ^b^	38

^a–c^ Different letters within a row are significantly different (*p* < 0.05). *n* = 4. Abbreviation: SEM, standard error of the mean; BHA, butylated hydroxyanisole.

**Table 6:** Profile of volatiles from cooked chicken breast meat with different levels of oregano oil at day 7.

Compounds	Control (None)	100 ppm Oregano	300 ppm Oregano	400 ppm Oregano	5 ppm BHA	SEM
	Total ion counts × 10^4^					
2-Propanone	2307 ^a^	2031 ^a^	1966 ^a^	2065 ^a^	1224 ^a^	251
2-Propanol	991 ^a^	909 ^a^	1008 ^a^	452 ^a^	951 ^a^	188
Hexane	47 ^ab^	172 ^ab^	0 ^b^	0 ^b^	209 ^a^	40
Heptane	160 ^a^	0 ^b^	0 ^b^	0 ^b^	132 ^a^	17
Acetaldehyde	90 ^a^	0 ^b^	0 ^b^	0 ^b^	0 ^b^	1
Propanal	297 ^a^	0 ^b^	0 ^b^	0 ^b^	0 ^b^	2
Pentanal	1153 ^a^	563 ^b^	305 ^bc^	297 ^bc^	254 ^c^	69
Heptanal	72 ^a^	0 ^b^	0 ^b^	0 ^b^	0 ^b^	2
Octane	322 ^ab^	137 ^b^	397 ^ab^	309 ^ab^	822 ^a^	128
Cyclohexane	127 ^a^	45 ^b^	45 ^b^	26 ^b^	184 ^a^	18
Hexanal	41262 ^a^	7887 ^b^	1252 ^c^	1215 ^c^	2095 ^bc^	1422
α-Pinene	0 ^c^	325 ^bc^	762 ^a^	378 ^b^	0 ^c^	85
Camphene	0 ^b^	258 ^b^	866 ^a^	503 ^ab^	0 ^b^	133
Limonene	0 ^c^	114 ^bc^	349 ^ab^	465 ^a^	0 ^c^	62
β-Myrcene	0 ^b^	335 ^ab^	640 ^a^	412 ^a^	0 ^b^	92
γ-Terpenene	0 ^b^	491 ^ab^	1326 ^a^	1020 ^a^	0 ^b^	200
Sabinene	0 ^c^	73 ^bc^	133 ^b^	355 ^a^	0 ^c^	23

^a–c^ Different letters within a row are significantly different (*p* < 0.05), *n* = 4. Abbreviation: SEM, standard error of the mean; BHA, butylated hydroxyanisole.
